# Control of Muscle Mitochondria by Insulin Entails Activation of Akt2-mtNOS Pathway: Implications for the Metabolic Syndrome

**DOI:** 10.1371/journal.pone.0001749

**Published:** 2008-03-12

**Authors:** Paola Finocchietto, Fernando Barreyro, Silvia Holod, Jorge Peralta, María C. Franco, Carlos Méndez, Daniela P. Converso, Alvaro Estévez, Maria C. Carreras, Juan J. Poderoso

**Affiliations:** 1 Laboratory of Oxygen Metabolism, University Hospital, Buenos Aires, Argentina; 2 Department of Clinical Biochemistry, School of Pharmacy and Biochemistry, University Hospital, University of Buenos Aires, Buenos Aires, Argentina; 3 Department of Medicine, University Hospital, University of Buenos Aires, Buenos Aires, Argentina; 4 Department of Human Biochemistry, School of Medicine, University of Buenos Aires, Buenos Aires, Argentina; 5 Burke Medical Research Institute, Cornell University, Ithaca, New York, United States of America; University of Parma, Italy

## Abstract

**Background:**

In the metabolic syndrome with hyperinsulinemia, mitochondrial inhibition facilitates muscle fat and glycogen accumulation and accelerates its progression. In the last decade, nitric oxide (NO) emerged as a typical mitochondrial modulator by reversibly inhibiting citochrome oxidase and oxygen utilization. We wondered whether insulin-operated signaling pathways modulate mitochondrial respiration *via* NO, to alternatively release complete glucose oxidation to CO_2_ and H_2_O or to drive glucose storage to glycogen.

**Methodology/Principal Findings:**

We illustrate here that NO produced by translocated nNOS (mtNOS) is the insulin-signaling molecule that controls mitochondrial oxygen utilization. We evoke a hyperinsulinemic-normoglycemic non-invasive clamp by subcutaneously injecting adult male rats with long-lasting human insulin glargine that remains stable in plasma by several hours. At a precise concentration, insulin increased phospho-Akt2 that translocates to mitochondria and determines *in situ* phosphorylation and substantial cooperative mtNOS activation (+4–8 fold, *P*<.05), high NO, and a lowering of mitochondrial oxygen uptake and resting metabolic rate (−25 to −60%, *P*<.05). Comparing *in vivo* insulin metabolic effects on gastrocnemius muscles by direct electroporation of siRNA nNOS or empty vector in the two legs of the same animal, confirmed that in the silenced muscles disrupted mtNOS allows higher oxygen uptake and complete (U-^14^C)-glucose utilization respect to normal mtNOS in the vector-treated ones (respectively 37±3 vs 10±1 µmolO_2_/h.g tissue and 13±1 vs 7.2±1 µmol ^3^H_2_O/h.g tissue, *P*<.05), which reciprocally restricted glycogen-synthesis by a half.

**Conclusions/Significance:**

These evidences show that after energy replenishment, insulin depresses mitochondrial respiration in skeletal muscle *via* NO which permits substrates to be deposited as macromolecules; at discrete hyperinsulinemia, persistent mtNOS activation could contribute to mitochondrial dysfunction with insulin resistance and obesity and therefore, to the progression of the metabolic syndrome.

## Introduction

The powerhouse of the cell, mitochondria are responsible for sustaining energy levels. However, a number of the new functions discovered for mitochondria in the past decade depend not on energy demand but on adjustments to respiration [1]–[2]. Critical reduction of respiration takes part as well in the mechanisms of prevalent illnesses; a reduction of mitochondrial activity and the decrease in energy expenditure contribute substantially to metabolic dysfunction in aging, insulin resistance and diabetes and conducts to lipid accumulation [3]. Of the many studies focused on insulin resistance and mitochondrial dysfunction in the last decade [4], few have critically examined mitochondrial activity in terms of alternation between complete substrate oxidation and storage as macromolecular deposits [glycogen or fat stores]. A mutual connection between decreased respiratory rate and the synthesis pathways was reported by Petersen *et al* who found that 40% reduction in mitochondrial oxidative and phosphorylation activity increased intramyocellular lipid content in elderly volunteers with severe muscle insulin resistance, compared with matched young control subjects [5]. Although lipid and glycogen accumulation further increases insulin resistance [6], a clear mechanism for reduction of mitochondrial oxidations with displacement of substrates to storage was not defined in this context yet.

With high affinity for O_2_, cytochrome *c* oxidase is responsible for the final transference of electrons to oxygen for reduction to water in mitochondria. In the last years, cumulative evidence shows that mitochondrial NO is a typical regulator that binds cytochrome oxidase at subunit II [7], competes with high-affinity for oxygen occupancy, and thus reversibly inhibits cytochrome oxidase and reduces respiration at nanomolar concentrations; at equal demands, physiologic *in vivo* oxygen utilization by mitochondria depends on the matrix NO/O_2_ ratio [8].

Although the diffusible NO yielded by cytosol nitric oxide synthase (NOS) isoforms modulates respiration *in* vivo [9], an advantage comes from the mitochondrial compartmentalization of NOS (mitochondrial nitric oxide synthase; mtNOS) [10] that directs NO flux vectorially to the matrical space. In particular, neuronal nitric oxide synthase [nNOSα] binds complexes I [11] and IV [12] through specific domains; most of the mitochondrial NO yield is abolished by targeted disruption of the nNOSα gene [13]. Likewise, activation of mtNOS and enhanced mitochondrial NO levels were previously demonstrated in normal rat development, hypothyroidism, cold acclimation, and hypoxia [14]–[16]. At reduced mitochondrial respiratory activity, mtNOS modulation should therefore be equally important for insulin effects. Matrix NO reduces oxygen uptake and ATP levels [17], thereby increasing the level of reduced equivalents and acetyl-S-coenzyme A (acetyl-CoA), the substrates for anaplerotic reactions and synthesis pathways stimulated by insulin. Moreover, in rat mitochondria, mtNOS is phosphorylated in an Akt-sensitive domain at Ser**^1412^**. The Akt/protein kinase B [PKB], a serine/threonine protein kinase with high homology for protein kinases A and C, was identified as the cellular homologue of the viral oncoprotein *v*-Akt (herein referred to as Akt). It is now clear that Akt1 and Akt2 have distinct functions, that both kinases are effectors of the insulin-PI3K pathway and that, Akt2 deficiency associates to a diabetes mellitus-like syndrome [18].

We therefore link here for the first time the mtNOS activity that results from nNOS confinement to muscle mitochondria with the respective rates of oxidative utilization of glucose or of glycogen synthesis induced by insulin. We postulate that insulin signaling activates Akt2, which not only activates the GLUT4 recycling pathway but also increases NOS activity in the organelles in an attempt to restrain mitochondria, thus favoring the replenishment of glycogen and fat energy stores. To that, we used a rat model of hyperinsulinemia with normal glycemia that allowed testing lasting effects of insulin on mitochondria. We succeeded in creating an *in vivo* comparative metabolic condition in which nNOS- and mtNOS-deficient muscles increase glucose and oxygen utilization and reciprocally restrict glycogen-synthesis providing molecular basis for understanding a NO-dependent progression of metabolic syndrome and type 2 diabetes.

## Results

### Insulin increases p-Akt2 in skeletal muscle mitochondria

**Figure 1:**
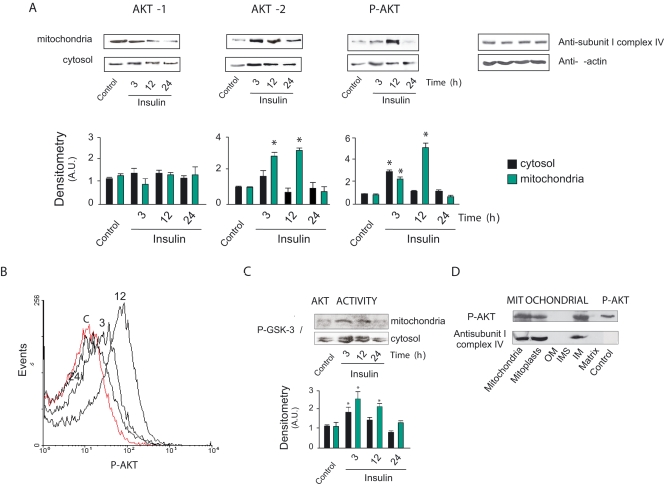
Insulin selectively increases p-Akt2 signal in skeletal muscle mitochondria. (A) Representative western blots of Akt1, Akt2, and p-Akt in proteins of rat skeletal muscle during hyperinsulinemic-normoglycemic status as achieved by s.c. administration of human insulin glargine plus oral sucrose (see Methods and Supporting Figure S2). Respective densitometries from 6–7 samples from separate experiments were obtained after digital image analysis and normalized to the actin band for cytosol fraction and to the complex IV subunit I band for mitochondria (Akt2 mitochondria: *F* = 39.03, *DF* = 23, *P* = 0.000; p-Akt cytosol: *F* = 111.8, *DF* = 23, *P* = 0.000; p-Akt mitochondria: *F* = 77.48, *DF* = 25, *P* = 0.000). Figure shows that after 12 hours increase of Akt2 and p-Akt2 is selectively confined to mitochondria. (B) Differential flow cytometry histograms of 100 µg of purified mitochondria isolated from controls (C) and insulin-treated animals were obtained with 1: 1000 fluorescent antibody anti-p-Akt in a mitochondrial population previously delimited with MitoTracker Red 580. Histograms show a kinetics similar to western blotting. (C) An Akt activity assay was performed three times by immunoprecipitation of mitochondria and cytosol proteins with anti-p-Akt antibody. After further immobilization, the proteins were detected with the anti-p-Ser21/9-GSK-3α/β antibody (1∶1000). The assay confirms the insulin-induced kinetics of p-Akt in the fractions as in (A) and (B) (Cytosol: *F* = 8.46, *DF* = 15, *P* = 0.003, Mitochondria: *F* = 8.15, *DF* = 15, *P* = 0.003). (D) Western blot shows submitochondrial localization of p-Akt at the inner mitochondrial membrane, as controlled by duplicate with an antibody specific for subunit IV of cytochrome oxidase. Data are mean±SEM.; * p<.05 respect to controls by ANOVA and Dunnett test.

Under fairly constant stimulation, insulin early increased the expression of Akt2 and phospho-Akt (p-Akt) in cytosol and mitochondria from skeletal muscle; thereafter, only mitochondrial Akt2 and p-Akt remained very high up to twelve hours after insulin (by about 4 and 6 folds, respectively). In contrast, insulin effects on Akt1 were poor and non-significant in the different fractions (Figure 1A). Flow cytometry of the isolated and purified mitochondria confirmed a net increase of p-Akt fluorescence, with a similar temporal kinetics to that detected by western blotting. (Figure1B). To test p-Akt2 activity under these experimental conditions, we measured the activation of the natural target of Akt, glycogen synthase kinase (GSK-3α/β), to its phosphorylated form (p-GSK-3α/β). Early in the experiment, p-GSK-3α/β peaked up in cytosol and mitochondria and those levels declined for the remainder of the experiment (Figure 1C). Submitochondrial fractionation demonstrated that p-Akt is localized at the inner mitochondrial membrane (Figure 1D). Selective increase of muscle Akt2 suggests the *in vivo* accumulation of mitochondrial p-Akt2 in the insulin-stimulated skeletal muscle.

### Insulin boosts nNOS activity in mitochondria

**Figure 2:**
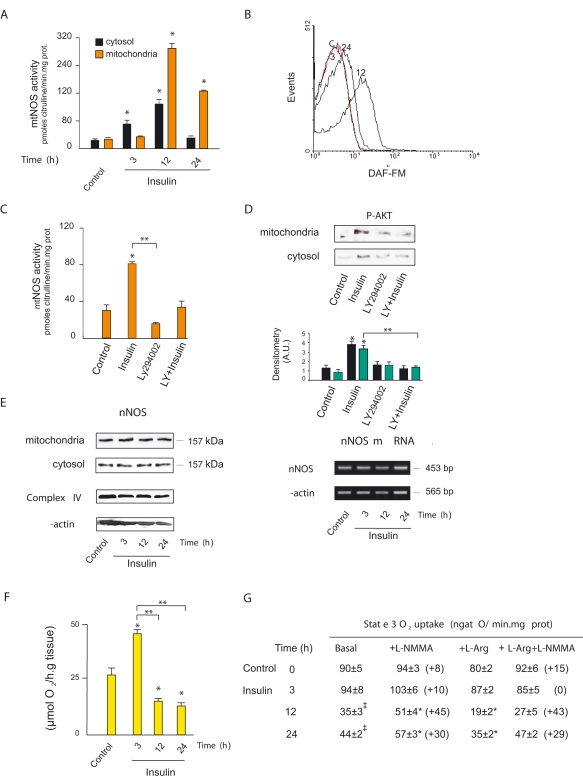
Insulin-PI3K signaling triggers high mitochondrial NOS activity. (A) Time-course of mitochondrial NOS activity after insulin administration measured by ^3^H-L-citrulline formation (n = 8; cytosol: *F* = 30.62, *DF* = 31, *P* = 0.000; mitochondria: *F* = 290.84, *DF* = 31, *P* = 0.000). (B) Matrix NO level appraised by DAF-FM fluorescence by flow-cytometry of isolated mitochondria; fluorescence was selectively analyzed in a mitochondrial population delimited by previous incubation with Mito Tracker Red 580 (C) mtNOS activity of mitochondria isolated from 200 mg of muscle slices preincubated by 30 min with 12 nM of insulin or 100 µM of LY29400 (PI3K-specific inhibitor) (*F* = 38, *DF* = 27, *P* = 0.000). (D) Representative western blot of muscle p-Akt2 distribution under the same conditions as in C. (cytosol: *F* = 14.75, *DF* = 15, *P* = 0.000; mitochondria *F* = 13.63, *DF* = 15, *P* = 0.000). (E) Expression of mtNOS and nNOS mRNA assessed by western blotting and RT-PCR, under the same conditions of Figure 1. (F) Polarographic determination of oxygen uptake of 100 mg of sliced gastrocnemius and extensor digitorum longus muscles obtained after insulin administration in Robinson buffer with 5 mM of glucose as substrate, pH 7.4 (n = 6; *F* = 18.32, *DF* = 22, *P* = 0.000). (G) State 3 oxygen uptake of mitochondria isolated from muscles was measured under the same conditions with malate-glutamate and ADP at 30°C in MSHE buffer, pH 7 and in the presence of L-NMMA, L-Arg alone or together with NOS inhibitor L-NMMA (n = 3–12; 12 h: *F* = 13.83, *DF* = 15, *P* = 0.000; 24 h *F* = 15.69, *DF* = 37, *P* = 0.000, *P<.05 *vs* respective basal values by Dunnett posthoc test; basal control and insulin-treated groups: *F* = 45.21, *DF* = 23, *P* = 0.000, ‡ *P*<.05 vs control by Dunnett posthoc test). In brackets, the percentage of variation respect to basal or L-arginine, respectively.

We next examine the relationship between the enhanced insulin-p-Akt2 pathway and the mitochondrial nNOS activity. At maximal mitochondrial p-Akt2, mtNOS activity proportionally increased by about 8 fold and remained high up to 24 hours (Figure 2A). Accordingly, high mitochondrial NO concentration was revealed by flow cytometry of isolated organelles with 4-amino-5-methyl-amino-2′, 7′-difluorescein diacetate (DAF-FM) (Figure 2B). To link NO to metabolic signaling, the muscle was challenged with insulin and the phosphatidyl-inositide_3_-kinase (PI_3_K) inhibitor LY294002, that resulted in a considerable 75% reduction in mitochondrial NOS activity and in blocking p-Akt accumulation in the organelles (Figure 2C and 2D). Taking into consideration that insulin elicits antagonistic responses, we searched for the optimal dose to obtain maximal NOS activity in mitochondria (Figure S1). Interestingly, remarkable effects on mtNOS were precisely achieved at half-dose insulin while they were less significant at the highest dose. Increase of NOS activity was not associated with changes in protein expression (Figure 2E), and other isoforms were not found in the organelles (not shown).

To quantify the inhibitory effects of NO on the electron transfer chain, we measured O_2_ uptake in muscle slices and mitochondria. Muscle O_2_ uptake peaked 3 hours after insulin administration (*P*<.05 respect control), but significantly decreased at 12–24 h (*P*<.05 either respect control or 3 h) (Figure 2F). In accord, at 12–24 h insulin-administered animals with maximal mtNOS activity had only 40–50% of the mitochondrial respiratory rate of control non-treated animals (*P*<.05) (Figure 2G). Likewise, in those periods O_2_ utilization was maximally restricted to 20–40% of the control values by supplementation of organelles with L-arginine. Dependence of NO was as well revealed by the fact that O_2_ inhibition was significantly relieved by the NOS inhibitor N-monomethyl-L-arginine (L-NMMA) (*P*<.05) (Figure 2G). To discern the effect of mtNOS in the different periods, we calculated the sum of opposite effects of NOS substrate and inhibitor on basal O_2_ utilization that determines the mitochondrial NOS functional activity on respiration (11):
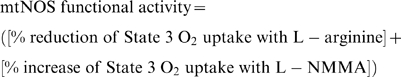
In accord to Figure 2G, mtNOS functional activity on respiration was maximal at 12 (91%) and 24 h (46%) post insulin treatment while at 3 h, it did not change respect to the control values (19%).

**Figure 3:**
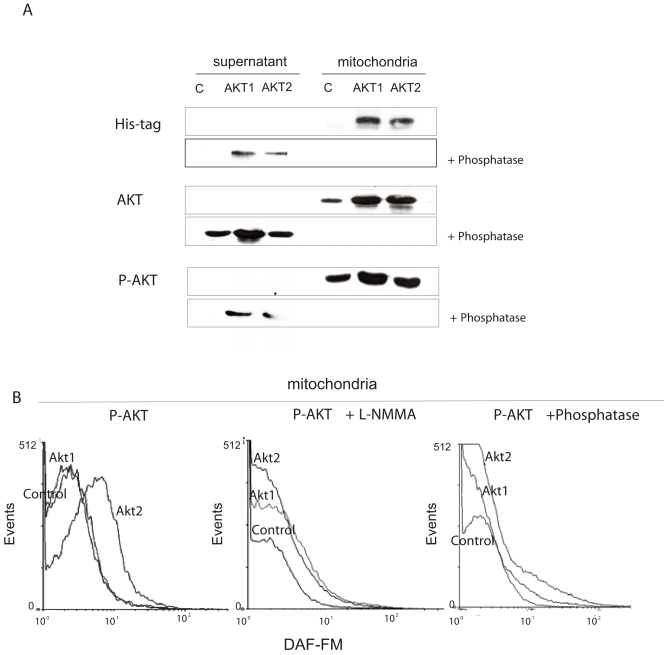
Phosphorylation allows translocation of Akt1 and Akt2 to mitochondria, but only p-Akt2 increases matrix NO. (A) Translocation of Akt1 and 2 was tested *ex vivo* in purified mitochondria isolated from rat muscle Mitochondria were suspended in MSHE buffer with NADH and ATP (pH7.4), incubated with recombinant His-tagged p-Akt1 and p-Akt2 proteins for 30 min at 30°C and centrifuged at 10000 *g* by 10 min to separate supernatant from mitochondrial pellet further resuspended in phosphate buffer saline (PBS) and incubated with proteinase *K* and compared with non-phosphorylated Akt. Western blots were performed with anti-His-tag (upper), anti-total Akt (middle) and anti-p-Akt antibodies (bottom). (B) To detect mitochondrial NO, flow cytometry histograms from isolated mitochondria (1 mg protein/ml) were obtained with DAF-FM in the same conditions of (A) and after incubating the organelles for 30 min a 37°C with recombinant phosphorylated and non-phosphorylated Akt1 and Akt2 alone or plus 3 mM L-NMMA. The histograms were obtained in a mitochondrial population previously delimited as in Figures 1 and 2.

To test whether these effects actually depend on p-Akt2 and whether p-Akt2 acts directly on NOS *within* mitochondria, we next incubated organelles from control muscle *ex vivo* with recombinant active Akt1 and Akt2 phosphorylated at Ser**^473^** and Thr**^308^**. In the presence of ATP and substrate, both phosphorylated Akt1 and Akt2 translocated to energized mitochondria and were detected even when incubated with proteinase *K*, whereas dephosphorylated Akt1 and Akt2 remained in the supernatant (Figure 3A); proteinase *K* removed non-translocated proteins that associate to the cytosol face of the outer mitochondrial membrane. In addition, when mitochondria charged with active Akt1 or Akt2 were exposed to DAF-FM, a peak of fluorescence was detected only in those treated with phosphorylated Akt2; this effect was not observed in mitochondria supplemented with dephosphorylated Akt and dissapeared by co-incubation with NOS inhibitor L-NMMA (Figure 3B). These results indicate that, at constant nNOS expression, subcellular distribution of p-Akt2 accounts for the temporal kinetics of nNOS activation in cytosol and mitochondria.

### Mitochondrial nNOS is phosphorylated by translocated p-Akt2: a cooperative kinetics

**Figure 4:**
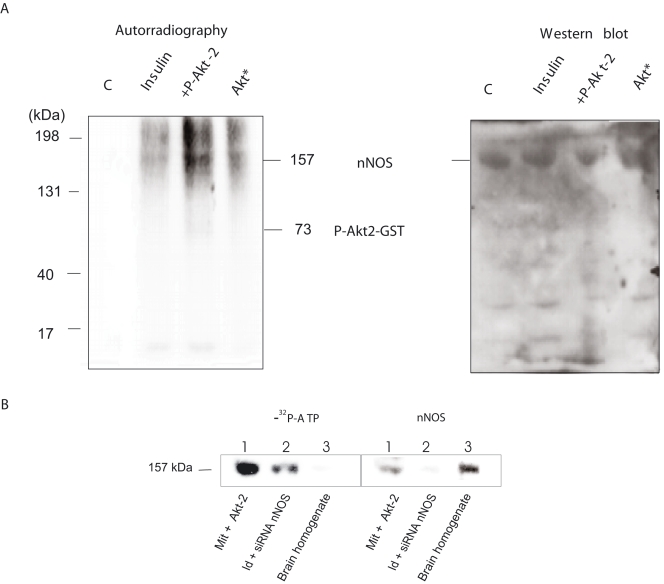
Insulin promotes mitochondrial NOS phosphorylation. (A) Representative autoradiogram of mtNOS phosphorylation in organelles isolated from the gastrocnemius muscle of animals administered with insulin at p-Akt2 peak concentration (12 h). Mitochondria (2 mg of protein) were incubated in 50 MSHE with 2 mM of cold ATP and 2 mM of NADH and 10 mC of γ^32^P-ATP with or without p-Akt2 or dephosphorylated Akt2 (*), as described in the legend of Figure 3. To the right, we show stable nNOS content in the same membrane of autoradiogram by western blot electrophoretic run. (B) Left panel: Autoradiogram of mitochondrial proteins isolated from the gastrocnemius muscle electroporated *in vivo* with empty pRNAT-U6.1/Neo vector (1) or with 10 µg of siARN nNOS (2) as compared with brain homogenate (3). Isolated proteins were processed as described in A; nNOS deficiency confirms insulin-induced mtNOS phosphorylation. Right panel shows a representative western blot of nNOS protein from the same electrophoresis.

**Figure 5:**
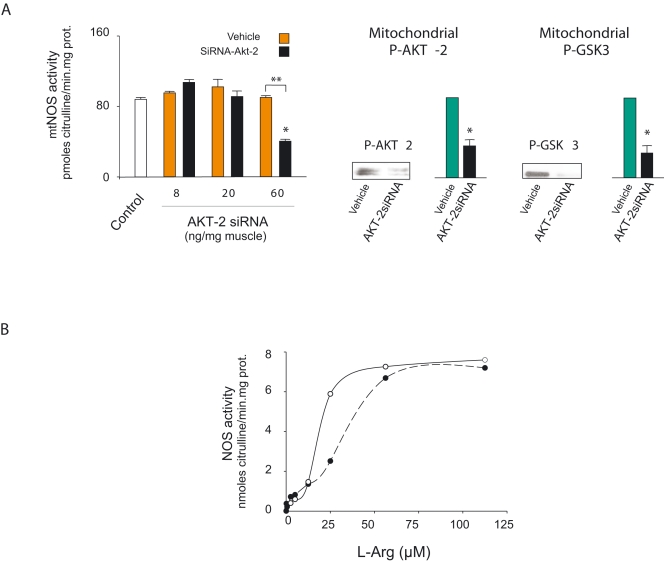
Translocated p-Akt2 phosphorylates and activates NOS through positive cooperative effects. (A) Dependence of mtNOS phosphorylation on p-Akt2 pathway is demonstrated by measuring the mtNOS activity of mitochondria isolated from the gastrocnemius muscle of rats treated with insulin at crescent siRNA Akt-2 (black bars), as compared with vehicle (grey bars), and muscle from non-inoculated animals (white) (n = 3; F = 66.55, *DF* = 11, *P* = 0.000; vehicle vs siRNA Akt-2 at 24 h: *t* = 18.05, *DF* = 4, *P* = 0.000). On the right, the signals of p-Akt and p-GSK-3 measured at 12 hours, which serve as controls, are controlled by western blotting and densitometry (n = 6; pAkt2: *t* = 8.4, *DF* = 10, *P* = 0.000; p-GSK3: *t* = 8.42, *DF* = 10, *P* = 0.001); The threshold for significance is the same as that described in Figure 2). (B) Kinetics of pure recombinant nNOS activity in a lipid phase with 10 µM of cholesterol and 20 µM of BH_4_; the dashed curve corresponds to non-phosphorylated nNOSα and the continuous curve to the cooperative shift of nNOSα phosphorylated by recombinant p-Akt2, as shown in intact mitochondria. Each point was done by duplicate/triplicate.

To confirm that the increase in mtNOS activity was a product of nNOS phosphorylation by translocated p-Akt, mitochondria from insulin-administered animals were supplemented with cold ATP and 15 mC γ^32^P-ATP and additional recombinant Akt2 before being subjected to autoradiography. In these conditions, a spontaneous signal of phospho-mtNOS was detected in skeletal muscle mitochondria with a high p-Akt2 charge (Figure 4A), that was further increased by exogenous recombinant p-Akt2. To confirm specificity, mitochondria were also isolated from nNOS-deficient muscle directly electroporated with nNOS siRNA. The right legs of rats were injected with nNOS siRNA and the left ones with an empty vector, and both were subjected to direct electroporation *in vivo*. These mitochondria exhibited low mtNOS content and therefore the Akt2-induced phosphorylation signal substantially decreased (Figure 4B). In accord, direct injection of Akt2 siRNA produced a dose-dependent inhibitory effect on mtNOS activity (60% at the highest dose), similar to the reduction of p-Akt2 and p-GSK3 (Figure 5A).

This effect suggests that the insulin-dependent increase in NOS activity is based upon the kinetic variations induced by Akt2-related enzyme phosphorylation in mitochondria (Figure 5A). To test this hypothesis, His-tagged nNOS was purified from bacteria transfected with cDNA in pCWori+ vector, and enzyme activity was assayed for phosphorylation in the presence of p-Akt2 and γ^32^PATP. The pioneer work by Fulton *et al* reported that Akt activates eNOS by phosphorylating Ser**^1179^** in transfected COS cells [19]. In their study, analogous phosphorylation of nNOS was observed only when the transfected construct had a myristoylation consensus site, indicating that attachment to membranes is also required for enzyme phosphorylation. Elfering *et al* reported that mtNOS is myristoylated and has only one Akt-sensitive domain (*RXRXXS/T*) at Ser**^1412^**, which has been reported to be phosphorylated in the Maldi/MS analysis [20]. We therefore measured K' for L-arginine in phosphorylated or non-phosphorylated recombinant nNOS in the presence of BH_4_ and cholesterol, as a lipid phase representative of membrane insertion (Figure 5B). The S1412G nNOS mutation, which simulates phosphorylation, increases the heme reduction rate and decreases NO yield and [21] electron transfer in the calmodulin-bound state. Here, a sigmoid kinetic curve was noted for both the phosphorylated and non-phosphorylated nNOS, but with different Hill slopes (3.7 and 1.9), findings that suggest a cooperative behavior (r^2^ = 0.99 and 0.97); in the lipid phase, p-Akt2 reduced K' for L-Arg from 19 to 11 µM.

### mtNOS is the final insulin/Akt effector that regulates muscle utilization of available glucose

**Figure 6:**
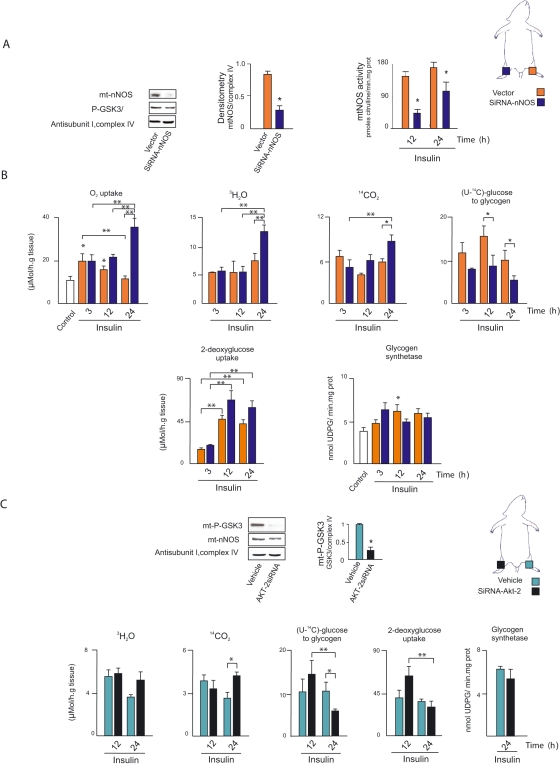
mtNOS Is a final insulin/Akt effector regulating muscle utilization of available glucose. (A) Representative western blot of disrupted and normal mtNOS, densitometries, and mtNOS activities of mitochondria isolated from right and left gastrocnemius of the same animal, 36 h after direct electroporation of respectively 10 µg of siRNA nNOS or empty pRNAT-U6.1/Neo vector to muscle, as described in Experimental Procedures. (n = 6; densitometry: *t* = 6.55, *DF* = 10, *P* = 0.000; activity at 12 h: *t* = 6.07, *DF* = 10, *P* = 0.000; activity at 24 h: *t* = 2.22, *DF* = 10, *P* = 0.05) (B) To test the interdependence of oxidative and intermediary metabolism as related to activation of mtNOS, metabolic studies including oxygen uptake, glycogen synthesis, complete glucose oxidation to CO_2_ and H_2_O, glucose uptake and glycogen synthase activity were performed in nNOS-silenced right gastrocnemius- and vector-administered left muscles of the same animal in parallel under the different conditions by radioactive methods (n = 6; vector: O_2_ uptake, *F* = 10.47, *DF* = 23, *P* = 0.000; ^14^CO_2_, *F* = 5.06, *DF* = 23, *P* = 0.02; (U-^14^C)-glucose to glycogen, *F* = 8.61, *DF* = 23, *P* = 0.006; 2-deoxyglucose uptake, *F* = 22.55, *DF* = 23, *P* = 0.000; glycogen synthase, *F* = 3.59, *DF* = 23, *P* = 0.032; with siRNAnNOS: O_2_ uptake, *F* = 13.78, *DF* = 23, *P* = 0.000; ^3^H_2_O, *F* = 17.34, *DF* = 23, *P* = 0.000; ^14^CO_2_, *F* = 4.25, *DF* = 23, *P* = 0.034; (U-^14^C)-glucose to glycogen, *F* = 8.08, *DF* = 23, *P* = 0.004; 2-deoxyglucose uptake, *F* = 12.90, *DF* = 23, *P* = 0.000; glycogen synthase, *F* = 3.62, *DF* = 23, *P* = 0.031). (C) Metabolic studies were performed by previous administration of crescent siRNAAkt2 or vehicle under conditions analogous to A and B (n = 3, p-GSK3: *t* = 7.47, *DF* = 4, *P* = 0.002; at 24 h, vehicle vs siRNAAkt2, ^14^CO_2_: *t* = −3.23, *DF* = 4, *P* = 0.032; (U-^14^C)-glucose to glycogen: *t* = 2.85, *DF* = 4 *P* = 0.05). The threshold for significance is the same as that described in Figure 4.*P<.05 *vs* controls or between nNOS or Akt2 silenced and not-silenced muscle samples; ** *P*<.05 between different times of insulin administration.

A cornerstone of this study is that, after energy replenishment, mitochondrial oxidations ought to be depressed to allow substrates to be deposited as macromolecules. We tested the contribution of mtNOS to the balance maintained between mitochondrial and intermediary metabolism, by injecting siRNA nNOS into rat gastrocnemius muscles with high nNOS content. This procedure reduced nNOS mRNA levels as well as protein expression and activity in mitochondria by approximately 50–77% in comparison to constant complex IV (Figure 6A). Under these experimental conditions and because of the decreased levels of matrix NO (Figure 2B), oxygen uptake became insensitive to insulin administration and remained high in the nNOS-silenced muscles. The increase of glucose uptake elicited by insulin administration was not modified by siRNA nNOS. Instead, siRNA nNOS markedly increased glucose utilization evidenced by complete oxidation to CO_2_ and H_2_O. Thus, at fixed insulin with similar glucose uptake and glycogen synthase activity, high oxygen utilization with siRNA nNOS was in line with high ^3^H_2_O and ^14^CO_2 _production rates, i.e., 50–100% higher in the right siRNA-treated muscle than in the left control (Figure 6B). No significant variations of metabolic rate were detected at low mtNOS activity. Interestingly, under these conditions the glucose used for glycogen synthesis was halved in the siRNA nNOS-treated muscle; at normal mtNOS content similar effects were observed when the Akt2 gene was silenced, reducing protein expression and phosphorylation of GSK-3α/β by 56% and 75%, respectively (Figure 6C). In accordance with previous reports, direct administration of siRNA Akt2 did not result in significant variations of glucose uptake or glycogen synthase activity, although the peak of glucose uptake decreased at 24 h in the deficient muscles (Figure 6C).

## Discussion

We herein demonstrate the modulatory connection between insulin signaling and mitochondrial function: insulin decreases the muscle oxidative rate *via* mitochondrial NO. We confirmed this finding using a number of different experimental approaches, showing that the effect relies on p-Akt2-selective phosphorylation of mitochondrial nNOS, after kinase translocation to the organelles.

A rapid translocation of p-Akt to the mitochondria and the phosphorylation of target GSK-3α/β by insulin-PI3K were previously reported by Bijur and Jope in a variety of cells [22]. It is confirmed here that phosphorylation is absolutely required for Akt translocation to muscle mitochondria and that, in this framework, both p-Akt1 and p-Akt2 can be translocated to the organelles *ex vivo* (Figure 3A). *In vivo* however, insulin only increases p-Akt2 probably because of high expression of SH2-phophatase that selectively dephosphorylates p-Akt1, but not p-Akt2 in skeletal muscle [23]. Moreover, solely translocated p-Akt2 and not p-Akt1 induced a robust generation of NO by mtNOS in the isolated mitochondria (Figure 3B). Considering that eNOS is activated in the caveolae by insulin/PI3K *via* p-Akt1, specific functional and topographic connections between NOS and Akt isoforms become evident. In this way, a recent study reported p-Akt2 phosphorylation of nNOS at Ser**^1412^** in synaptosomes, suggesting the functioning of this mechanism in different organs and subcellular fractions [24].

**Figure 7:**
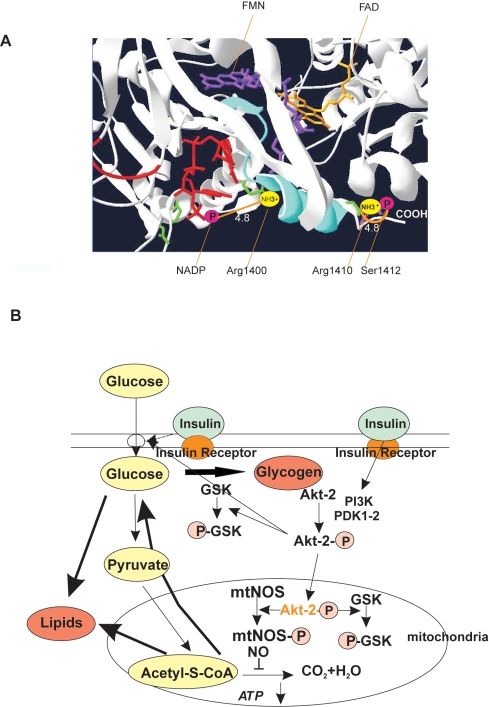
Scheme of nNOS activation and the modulation of muscle metabolism. (A) A pendulous movement of the nNOS C-terminal tail (in blue) by opposed electrostatic interactions is suggested to explain cooperative positive modulation of nNOS phosphorylated by p-Akt2; nNOS (gi: 16258810) was represented with Pymol (DeLano Scientific, Palo Alto, CA, USA). (B) Scheme of the modulation of mitochondrial respiration and glucose metabolism by the Akt2/nNOS couple in rat skeletal muscle.

The phosphorylation of nNOS/mtNOS by p-Akt2 involves cooperative effects centered at the C-terminal tail that contains the Akt motif (Ser**^1412^**; Figures 4B and 7A). Although structural changes of phosphorylated nNOS are not defined yet, it is worth noticed that C-terminal tail participates in the regulation of nNOS activity. Allosteric inhibition of electron transfer from NADPH to FAD and poor NO release at low Ca^2+^-calmodulin level result from electrostatic interactions between acidic negative charges of 2′phosphate of NADP and positive charges of basic Arg**^1400^** [25]. On this basis, we surmise that p-Akt2 activation of nNOS/mtNOS (Figures 4 and 5) promote opposed electrostatic interactions between Ser**^1412^** and Arg**^1410^** that, in the lipid phase, equilibrates the oscillation of the C-terminal tail to cooperatively increase the NO production rate (Figure 7A).

Different approaches confirmed in this study that phosphorylation of muscle mtNOS increases mitochondrial NO yield by several folds and reduces systemic O_2_ utilization. We show here as well that the temporal activation of mtNOS and the resulting decline of mitochondrial O_2_ uptake *via* NO drive the muscle insulin-transition from glucose oxidation to glycogen deposition. In absence of NO or, in nNOS-silenced muscles, mitochondrial O_2_ uptake is completely released leading to a preferential oxidation of glucose to CO_2_ and H_2_O; in these conditions, prevalent oxidation of glucose reflects the respective *Km* of regulatory enzymes like cytochrome oxidase for O_2_ and pyruvate dehydrogenase complex for acetyl-CoA, at least one order of magnitude lower (10^−7^–10^−8^ M, ref 26), than that of glycogen synthase for glucose (10^−5^ M, ref 27). In contrast, in the presence of NO, glucose utilization is delivered to glycogen synthesis. These results therefore support the notion that a discrete inhibition of the respiratory chain as produced by NO is required to reduce oxidative rates and to allow glycogen or fat deposition (Figure 7B).

At ten-fold higher insulin dose, maximal mtNOS activation was reduced by a half (Figure S1). This fact reminds that insulin activates other mitochondrial reactions as well; *i.e.,* early activation of pyruvate dehydrogenase complex through insulin-inhibition of pyruvate dehydrogenase kinase (PDK4) could transiently compete with NADH/NADPH availability for NOS in skeletal muscle mitochondria [28]. In this study, effects at low and high insulin concentrations thus explain: a) lack of stimulation and null mtNOS activity in muscle at initial blood insulin peak (3 hours); b) high respiratory rate at peak insulin level with null mtNOS (Figure 2G); c) decreased respiratory rate at stable insulin concentration with full mtNOS and, d) physiological alternation of insulin effects promoting complete glucose oxidation or glycogen synthesis (Figures 2 and 6). The increase or decrease of oxygen consumption was previously reported to occur in muscle at respectively high or low insulin concentration [29].

The presented data confirm insulin as a normal regulator of oxidative phosphorylation rate in skeletal muscle but as well they could explain cardinal features of the progression of metabolic syndrome and obesity to type 2 diabetes. Zucker Fatty (ZF) rats with a leptin receptor defect exhibit obesity, insulin resistance with hyperinsulinemia and normal glycemia that progress to type 2 diabetes in 6–12 weeks (4); ZF rats have severe mitochondrial inhibition with low oxidation of palmitic acid to CO_2_ [30]. Moreover, leptin-deficient *ob* ^−^/*ob* ^−^ obese mice exhibit insulin resistance and obesity associated to hyperinsulinemia with mitochondrial inhibition [31]. Preliminary results of our group demonstrated that *ob^−^*/*ob^−^* mice with metabolic syndrome have increased expression of mtNOS and high mitochondrial NO yield and that mitochondrial inhibition is released by NOS inhibitors or by leptin infusion, both decreasing NO yield in the organelles [32]. As in genetic experimental models of metabolic syndrome, the term mitochondrial dysfunction has been referred in humans as to a condition with decreased oxidation rate and ATP synthesis [33] and, in this context, reduced respiration was reported to contribute to glycogen or fat deposition [3] and can be independent of insulin resistance [34]. Considering the metabolic syndrome as accompanied by insulin resistance with prolonged hyperinsulinemia, such a mitochondrial dysfunction appears to be analogue to the persistent inhibitory effects on mitochondrial respiration demonstrated by the presented data at intermediate insulin concentration.

Our findings therefore suggest that the mechanisms for progression to severe mitochondrial dysfunction in type 2 diabetes are contributed by NO-dependent mechanisms. We reported that NO markedly increases superoxide anion yield in rat skeletal muscle mitochondria by inhibiting electron transfer at Complexes I and III [35], finally damaging Complex I [36]. Increased mitochondrial superoxide anion was related to hyperglycemia and insulin resistance, as well as to persistent mitochondrial dysfunction [37]. In the presence of superoxide anion plus nitric oxide, 3-nitrotyrosine was found as a marker of nitration of mitochondrial proteins in the *ob* ^−^/*ob* ^−^ metabolic syndrome [31] and in experimental and clinical diabetes [38]. Excessive NO leads both to nitrosylation and inactivation of the insulin receptor and Akt, increasing insulin resistance [39]. A number of different NO-based mitochondrial mechanisms can contribute to insulin resistance as well. First, an increase of ADP/ATP ratio due to inhibition of electron transfer [17] can restrict the phosphorylation of insulin receptor substances (IRSs) and the translocation of GLUT4 to the cell membrane. Second, accumulation of NADH due to NO-dependent complex I inhibition can negatively modulate glycolisis and the Krebs cycle [40]. Finally, nitric oxide stimulates mitochondrial biogenesis by increasing PGC 1α coactivator and thus could compensate mitochondrial dysfunction in the early metabolic syndrome, while reduced biogenesis with further decay of mitochondrial population has been reported in already established type 2 diabetes [41].

From these perspectives, an additional explanation is required for progression of diabetes and tissue damage in hypoinsulinemic status, like type I diabetes, Akt2 deficiency or nNOS knocked out mice. In contrast to activation of insulin-NOS pathway in mitochondria, in these models progressive tissue and mitochondrial damage should not depend on an increased ADP/ATP ratio; it is worth noticed that Akt2 has extramitochondrial effects on GLUT4 and facilitates *per se* the mechanism of glucose uptake. However, it is apparent as well that weight loss in low-insulin diabetes or severe lypoatrophy in hypoinsulinemic Akt2-deficient mice could occur because of the lack of NOS activation, leading to uncontrolled mitochondrial respiration and to high oxidative stress in the organelles with a marked reduction of glycogen content [18].

We conclude that increase of NO in the mitochondrial compartment by insulin is a powerful physiological resource to properly adjust muscle O_2_ utilization, while persistence of this mechanism in hyperinsulinemic states is harmful and could contribute to diabetic mitochondrial dysfunction.

## Materials and Methods

### Animals and treatments

Animals were housed in a temperature-controlled room, provided food, and subjected to a 12:12 dark/light cycle. National Institutes of Health criteria for animal research were followed after approval by the University Hospital.

To achieve a non-invasive hyperinsulinemic-normoglycemic clamp, male Wistar rats [250–300g body wt] were subcutaneously inoculated with long-acting human analogue insulin glargine (Supporting Figure S2, 42–44) or NaCl 0.9% and skeletal muscle was excised at 3, 12 and 24 hours. Insulin glargine, with its three modifications to human insulin (Gly**^A21^**, ,Arg**^B31^**, Arg**^B32^**) is a stable molecule that is soluble in slightly acidic conditions and precipitates in the neutral pH of subcutaneous tissue. Because of these properties, absorption of insulin glargine is delayed and the analogue provides a fairly constant, basal insulin supply without peaks in plasma insulin levels for approximately 24 hours, similar to that achieved by a continuous subcutaneous insulin infusion (Figure S2). During the 24-hour period, active metabolites sustain insulin effects after the decay of the hormone. Animals were allowed to drink water with 5% sucrose *ad libitum* to avoid hypoglycemia (Figure S2). Without glucose intake, insulin glargine results in hypoglycemia 2–4 h after administration (−15 to 30% of basal values; 45). Plasma insulin levels were followed independently of endogenous hormone with a specific human immunoassay (IMX Microparticle Enzyme Immunoassay, Abbott Labs, Ramsey, MN). Because the cross reactivity between insulin glargine and human insulin is approximately 60%, thus resulting in underestimation of its level in the immune assay, obtained values were multiplied by 1.8 [43]. All experiments were carried out in rat extensor digitorum longus and gastrocnemius red-mixed muscles with appropriate mass (∼0.6 g per muscle); adult rat muscle is comprised of approximately 4–50% slow oxidative fibers [I] and 20–40% fast oxidative fibers [IIa] with a large mitochondrial population, high glycogen content, and concomitantly, a high level of nNOS activity [46].

### Akt/insulin Signaling Pathway and Nitric Oxide in Muscle Mitochondria

Muscle mitochondria were isolated from homogenized muscles by differential centrifugation and further purified with Percoll gradients. After purification, the mitochondrial fraction has no more than 10% activity of cytosol lactic dehydrogenase activity or 5% of calreticulin from SRE (western blot) while cytosolic fraction did not express complex I proteins or 39 kDa subunit I of cytochrome oxidase (western blot). Cytosol and mitochondrial expression of nNOS, p-Akt, Akt1, Akt2 and p-GSK-3 α/β was assessed by immunoblotting with specific antibodies and by detecting variations of NO and p-Akt, by flow-cytometry in freshly isolated mitochondria.

To analyze effects of PI3K pathway on mtNOS, muscles were dissected out and rapidly cut into 20 to 30 mg strips and incubated in a shaking water bath at 30°C for 30 min into a 25 ml flask containing 3 ml of oxygenated Robinson buffer supplemented with glucose and 0.1% BSA; strips were homogenized and mitochondria were obtained. Flasks were gassed continuously with 95% O_2_-5% CO_2_ throughout the experiment.

NOS activity was followed in cytosol and purified mitochondria by conversion of [^3^H]-L-arginine to [^3^H]-L-citrulline. To detect variations of NO and p-Akt by flow-cytometry, freshly isolated mitochondria were previously incubated for 30 min at 37°C with fluorescent anti-p-Akt or 10 µM of DAF-FM and 0.5 µM of MitoTracker Red 580 in PBS 1X. Fluorescence intensity was measured using an Ortho Cytoron Absolute Flow Cytometer [Johnson and Johnson, Raritan, NY]. Submitochondrial p-Akt localization was studied by western blot after hypotonic disruption and differential centrifugation; p-Akt activity was determined with a commercial kit from Cell Signaling (Beverly, MA.). [^3^H]-L-citrulline assay was done in 50 mM of potassium phosphate buffer, pH 7.5, in the presence of 100 µM of L-arginine, 0.1 µM of [^3^H]-L-arginine (NEN, Boston, MA.), 0.1 mM of NADPH, 0.3 mM CaCl_2_, 0.1 µM of calmodulin, 10 µM of tetrahydrobiopterin, 1 µM of FAD, 1 µM of FMN, 50 mM of L-valine and 1 mg/ml of protein [11]. Ca^2+^-dependent specific activity was calculated by subtracting the remaining activity in the presence of the NOS inhibitor L-*N^G^*-methyl-L-arginine (5 mM of L-NMMA) or 2 mM of EGTA ^3^H arginine assay. NOS activity was measured as the number of mitochondria isolated from rat muscle injected with insulin and from muscle incubated with insulin or LY29400 (PI3K-specific inhibitor). Muscles were dissected out and rapidly cut into 20- to 30-mg strips and incubated in a shaking water bath at 30°C for 30 min before being transferred into a 25-ml flask containing 3 ml of oxygenated Krebs-Ringer bicarbonate buffer (KRB) supplemented with 8 mM of glucose, 32 mM of mannitol and 0.1% BSA. Flasks were gassed continuously with 95% O_2_-5% CO_2_ throughout the experiment. To measure the mtNOS activity, muscle mitochondria were isolated and purified after incubation, as described previously.

Total muscle RNA was extracted with Trizol, and RT-PCR was performed under standard conditions.

### Translocation of Akts to mitochondria

Isolated organelles at 1 mg/ml were purified with percoll gradients and incubated *ex vivo* with pure recombinant active Akt1-GST or Akt2-GST (100 units/ml) (Cell Signaling) dually phosphorylated at Thr-308 and Ser-473 or with isoforms dephosphorylated with acid phosphatase and co-incubated with the NOS inhibitor L-NMMA. Mitochondria were then centrifuged and separated from supernatant, co-incubated with 50 µg/ml proteinase *K* and DAF-FM, and subjected to flow cytometry to assess fluorescence intensity. To allow translocation of Akt2 to the inner mitochondrial membrane [47], mitochondrial membrane potential was sustained by co-incubating the isolated organelles with 2 mM NADH and 2 mM ATP without Mg_2_Cl.

### nNOS phosphorylation by p-Akt2

NOS phosphorylation was evaluated in a mitochondrial free assay with γ^32^P-ATP and cold ATP in MOPS buffer alone or together with active human recombinant Akt2 phosphorylated at Ser**^473^** and Thr**^308^**. Recombinant His-tagged nNOS protein was obtained by magnetic field from *E. coli* transformed in a pcWori+ vector containing the respective cDNA. Kinetics parameters were assessed with GraphPad Software (San Diego, CA); K' = [V*max*×[L-Arg]^h^/V]-[L-Arg]^h^, where h is the calculated Hill coefficient.

### Oxygen uptake

Oxygen uptake was measured at 220 µM of O_2_ in a standard polarograph (IQUIFYB-MADEIC, Buenos Aires, Argentina) in sliced muscle with Robinson buffer or in isolated mitochondria with MSHE buffer with 6 mM of malate plus 6 mM of glutamate, with or without 0.2 mM of ADP, 0.3 mM of L-arginine or 3 mM of L-NMMA.

### In vivo muscle siRNA electroporation

Animals were briefly anesthetized with sodium thiopental [40 mg/kg weight, intraperitoneal], and gastrocnemius muscles were percutaneously treated with one proximal and one distal injection of siRNA nNOS (10 µg/per muscle] [GenScript, NewJersey, N.Y.) (right leg) or empty pRNAT-U6.1/Neo vector (left leg) or with Akt2 siRNA from Santa Cruz Biotechnology (Santa Cruz, CA) (right leg) or vehicle (left leg), in parallel. Each injection was performed in 5 µl free-RNAase solution, slowly in 15–20 s with a Hamilton syringe with a 22 G needle. Around 30 s after injection, electric pulsation was applied. For pulse delivery to muscle, two stainless steel connectors were applied on the needles implanted in muscle. Therefore, the needles were used as electrodes and connected to the generator; in these conditions the depth of insertion was about 0.5 cm and the gap between the two needles was 0.9 cm. The muscle were then electropulsed with selected parameters using the Gene Pulser II Electropulsator (Bio-Rad Laboratories,USA).Voltage (120V), pulse duration (20 ms) and frequency of pulses (1Hz) were all preset on the electropulsator. A train of eight pulses was delivered. [46]. The sequences of nNOS siRNAs were designed on the structure of rat nNOS g.i.: 16258811 and are provided in Figure S3.

### Metabolic studies

Thirty six hours after electroporation, insulin was administered at appropriate times and finally, animals were anesthetized and gastrocnemius muscles were removed, weighed, and washed with NaCl (0.9%). Finally, muscles were excised and minced in 0.1-mm layers with a Thomas slicer and 180–200 mg of tissue were placed into three different test tubes containing Krebs-Ringer bicarbonate buffer with 2% BSA pH 7.2 (250 μl/100 mg of tissue) and gassed with O_2_:CO_2 _[95:5 vol/vol] for five minutes at 37°C. To measure glycogen synthesis and complete glucose oxidation to CO_2_ and H_2_O, muscle was supplemented with 0.5 μCi/ml deoxy-D-glucose,2 [1,2^3^H[N]]-[25.0 Ci/mmol], 4 μCi/ml glucose, D-[^14^C[U]]-[250 mCi/mmol] and 4 μCi/ml glucose D-[5-^3^H[N] ]-[10.2 Ci/mmol]. Radioactives were from Perkin-Elmer Life and Analytical Sciences, Boston, MA, USA.

Determinations were done after 12 hours of fasting; to maintain both appropriate light-dark cycle and fasting, samples for glycogen synthesis after 12 hours of insulin administration were obtained at 6:00 PM and for glucose oxidation at 3:00 and 11:00 AM.

To measure glucose uptake, muscle was placed separately in the appropriate buffers and supplemented with radioactive deoxyglucose [48]. Glycogen synthase activity was measured using the method developed by Leloir and Goldemberg [49].

### Statistical analysis

Data were compared with ANOVA and the Dunnett *post hoc* test or the two-tailed Student's *t* test as appropriate; significance was accepted at *P*<.05.

## Supporting Information

Figure S1 Muscle mtNOS activity at different doses of insulin glargine. Each value represents separate experiments from animals with insulin (closed circles) and without insulin (closed squares) (n = 6, *P<.05 by ANOVA and Dunnett test). Strong activation of mtNOS selectively occurs at half-maximal insulin dose.
(0.17 MB EPS)

Figure S2 Effects of long-lasting insulin in rats. (A) A non-invasive hyperinsulinemic normoglycemic clamp was achieved in rats by subcutaneous administration of 0.1 U/Kg of insulin glargine and 5% oral sucrose ad libitum; after an initial peak, level of insulin+metabolites remains remarkably stable by 20 hours. (B). The rate of sucrose intake of animals with or without (C) insulin (white bar), at the same dose but at different times (dark bars); *P<.05 respect control by ANOVA and Dunnett test).
(0.24 MB EPS)

Figure S3 Sequence of hairpin siRNAs designed for rat nNOS (gi: 16258810). siRNAs were cloned in vector pRNAT-U6.1/Neo with BamH I and Hind III restriction enzymes; in red, poly A tail. siRNA # 1 was preferentially used in the in vivo studies because of its maximal inhibitory effect.
(0.33 MB EPS)
